# Role of Exendin-4 Functional Imaging in Diagnosis of Insulinoma: A Systematic Review

**DOI:** 10.3390/life13040989

**Published:** 2023-04-11

**Authors:** Marko Magdi Abdou Sidrak, Maria Silvia De Feo, Ferdinando Corica, Joana Gorica, Miriam Conte, Luca Filippi, Laura Evangelista, Giuseppe De Vincentis, Viviana Frantellizzi

**Affiliations:** 1 Department of Radiological Sciences, Oncology and Anatomo-Pathology, Sapienza University of Rome, 00161 Rome, Italy; 2 Department of Nuclear Medicine, Santa Maria Goretti Hospital, 04100 Latina, Italy; 3 Nuclear Medicine Unit, Department of Medicine (DIMED), University of Padua, Via Giustiniani, 35128 Padua, Italy

**Keywords:** exendin-4, PET, SPECT, insulinoma

## Abstract

Background: Insulinomas are the most common neuroendocrine neoplasms of the pancreas. Diagnosis is made through patient clinical presentation with hypoglycemia symptoms and imaging, such as EUS, CT, MRI, and functional imaging. Exendin-4 PET/CT (and SPECT/CT) is a new prominent radiotracer developed to image insulinomas. The aim of the study is to evaluate whether exendin-4 imaging is a useful tool in imaging for insulinoma patients when other imaging methods do not reach them. Methods: MEDLINE research conducted on PubMed, Scopus, and Web of Science gathered a total of 501 papers. Studies that evaluated exendin-4 SPECT and PET in insulinoma patients were screened and assessed through QUADAS-2 for risk of bias and applicability concerns’ assessment. Sensitivity, specificity, and accuracy were reported when available. Results: A total of 13 studies were deemed eligible for a QUADAS 2 review. Studies included ranged from 2009 to 2022. The most-used tracer was ^68^Ga-DOTA-exendin-4 in PET and ^111^In-DTPA-exendin-4 in SPECT. Exendin-4 labeled with ^99^mTc was also reported. The QUADAS-2 risk of bias assessment was overall low, with some unclear reports in the reference and index domains. Only two domains were at high risk of bias because of an explicated non-blind imaging review. Applicability concerns for bias were low in all domains. Reported sensitivities ranged from 95% to 100% and specificities from 20% to 100%. Conclusions: exendin-4 imaging is a sensitive functional imaging tracer in both SPECT and PET applications, especially in suspicion of benign insulinomas located where endoscopic ultrasound cannot reach, being more sensitive than morfostructural imaging.

## 1. Introduction

Insulinomas are insulin-secreting neuroendocrine neoplasms (NEN), with a very low incidence of 1–4 cases per million people per year [1]. Nonetheless, it is the most common NEN of the pancreas [2]. Benign insulinomas account for the vast majority, while the estimation for malignant insulinomas accounts for about 6% of all insulinomas [3]. Other authors report 90% as a rate of benign insulinomas, distributed in the entire pancreas; usually, 90% of them are solitary and in intrahepatic sites, with diameters less than 2 cm. Extrahepatic lesions are very rare and mainly found in the duodenal wall [1]. Diagnosis is carried out at the median age of 47 (range 8–82) years, with a male to female ratio 1.4:1. Symptoms at presentation are vague and heterogeneous and can differ both in time and in manner within the same patient; indeed, they can be autonomic, caused by episodes of insulin secretion determining tremors, palpitations, and diaphoresis, as well as by inducing hypoglycemia, characterized by confusion, personality and behavioral changes, visual impairment, seizures, and coma. Confirmation of insulinomas used to be made via the Whipple triad, consisting of hypoglycemia at symptom presentation, symptoms compatible with hypoglycemia, and relief after glucose administration [1]. Insulinomas arose frequently in MEN-1 patients (a rare syndrome with predisposition to primary hyperparathyroidism, neuroendocrine tumors (NETs), and pituitary adenomas) [4]. Causes of hypoglycemia can be multiple, from excessive glucose use or loss, or reduced glucose production. Hypoglycemia can also be postprandial (due to congenital enzyme deficiency, galactosemia, post-gastric resection) or postabsorptive (due to drugs such as insulin, sulfonylureas, or alcohol, as well as due to sepsis, hepatic or renal failure, and Addison’s disease) [5]. Imaging techniques to localize insulinoma lesions are as follows: abdominal ultrasonography (US), endoscopic ultrasonography (EUS), computed tomography (CT), magnetic resonance imaging (MRI), and molecular imaging (i.e., PET and SPET). US has recently improved its sensitivity, with reports of up to 94% for hypoechoic rounded lesions with distinct margins. However, the identification of lesions in the distant tail of the pancreas still remains a limitation of US due to its intrinsic characteristics [6]. Therefore, the sensitivity increases for tumors of the head of the pancreas and decreases in the tail and in extra pancreatic lesions [7]. Contrast-enhanced CT (ceCT) has the advantage of being operator independent compared with US, plus it allows exact localization of the lesion and its relationship with the surrounding structures. Insulinomas are usually greatly vascularized, thus showing enhancement during the arterial phase of a CT scan. Other less common appearances can occur, such as hypovascular or hypodense post-contrast lesions, cystic masses, and calcified masses that are more likely to be malignant than benign. A recent study has reported that multidetector CT scanners can visualize up to 94.4% of insulinoma lesions [1]. MRI is also proving to be a good imaging device for insulinoma detection, with a high sensitivity. Insulinomas appear as hyperintense in T2 sequences and as hypointense in T1 sequences [8]. Molecular imaging provides somatostatin receptor imaging (SSTR), being able to localize the lesion based on the expression of the receptor binder. Octreotide analogues show high affinity for SSTR2 and different degrees of affinity for SSTR3, SSTR4, and SSTR5 [9]. One-third of insulinomas do not express SSTR2 or SSTR5 and therefore an octreotide scan results in a false-negative finding [10,11]. PET can be acquired by using alternative tracers such as fluorodeoxyglucose (FDG), dihydroxyphenylalanine (DOPA), 5-hydroxytryptophan (5-HTP), and DOTA analogs [10]. Lately, exendin-4 has been used for functional imaging in both PET and SPECT. It is a peptide made of 39 amino acids, taken from Gila monster saliva and similar in structure to the glucagon-like peptide-1 (GLP-1) mammalian incretin as it binds to GLP-1R on β pancreatic cells [12]. GLP-1 receptors are present mainly in the pancreas, but also in the stomach, in parafollicular C cells, and in blood vessels. Overexpression of this receptor has been demonstrated in benign insulinomas, establishing new GLP-1R target imaging in these tumors [13]. The current gold standard, selective arterial calcium stimulation (CaS) and venous sampling studies (VaS), has a higher sensitivity (85%) but a limited ability in insulinoma localization. The inclusion of noninvasive approaches in the diagnostic algorithm for the diagnosis is strictly needed for replacing, or providing additional information on, the invasive procedures [14]. The aim of this review is to gather available studies on the exendin-4 agent for functional imaging by evaluating the state of the art in the insulinoma diagnosis and to compare it with morpho-structural imaging. The existing literature on exendin-4 imaging techniques includes several studies that have evaluated its diagnostic accuracy for insulinomas using various imaging modalities. However, limitations such as selection bias, small sample size, heterogeneity in study design, and patient populations are present. A QUADAS2 review of exendin-4 imaging techniques will provide a systematic and comprehensive evaluation of the diagnostic accuracy and reliability of exendin-4 imaging for insulinomas and help to identify potential sources of bias and variability across studies. By evaluating the quality of the included studies based on predefined criteria, a QUADAS2 review can provide a more rigorous and unbiased assessment of the diagnostic accuracy of exendin-4 imaging for insulinomas. Additionally, a QUADAS2 review can provide insights into the optimal use of exendin-4 imaging for diagnosis and management of insulinomas and can inform the development of clinical guidelines and recommendations. Overall, while the existing literature on exendin-4 imaging provides valuable insights into its diagnostic accuracy, a QUADAS2 review can provide a more comprehensive and rigorous evaluation that can enhance the reliability and validity of exendin-4 imaging for insulinomas.

## 2. Materials and Methods

### 2.1. Search Strategy and Study Selection

The systematic review was done according to the Preferred Reporting Items for Systematic Reviews and Meta-Analyses (PRISMA) guidelines. Publications included papers from January 1993 to December 2022. The research was conducted on Pubmed, Web of Sciences, and Scopus. The following keywords were used as research terms: “exendin-4” and “pet” or “spect” or “exendin-4” and “insulinoma” and “pet” or “spect”. Articles not in English or dosimetric studies, reviews, and case reports were excluded from the evaluation. Clinical studies that compared exendin-4 PET/CT with other imaging techniques were also assessed.

### 2.2. Data Extraction and Methodological Quality Assessment

For each eligible study, sensitivity, specificity, accuracy, number of patients, year of publication, and country, as well as authors’ generalities, were retrieved for the included clinical studies. Quality Assessment of Diagnostic accuracy Studies-2 (QUADAS-2), a mean that is frequently used for systematic review of diagnostic accuracy by the Agency for Healthcare Research and Quality, the Cochrane Collaboration, and the U.K. National Institute for Health and Clinical Excellence was used for the systematic review of diagnostic accuracy by submitting each eligible article through the signaling questions for the “Risk of Bias” and the “Applicability Concern Assessment” [15,16,17].

## 3. Results

**Figure 1:**
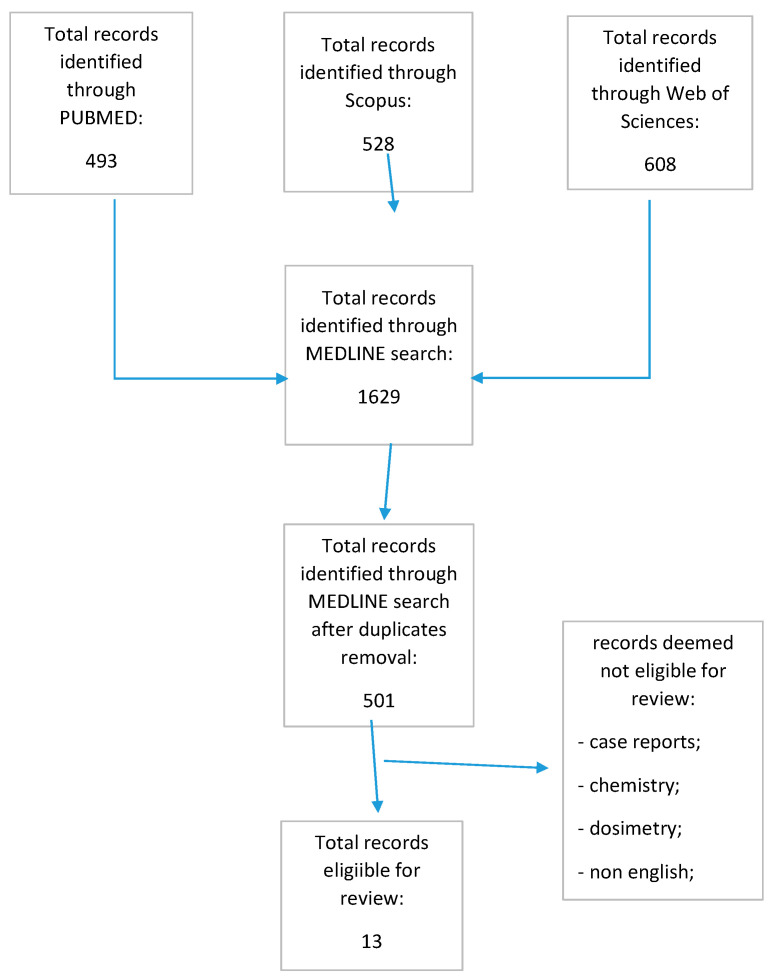
Prisma flow chart.

The MEDLINE search conducted on three different sources identified a total of 501 records after duplicate removal. Again, after skimming the retrieved records and eliminating the studies that were unrelated, a total of 13 studies dating from 2009 to 2021 were deemed eligible for review (Figure 1).

The QUADAS-2 assessment for risk of bias was overall low (34/52, Table 1 and Table 2). Unclear domains were 17/52 in the index and reference tests, as the authors did not explicate whether radiologists and pathologists were blinded to the reference and the index, respectively. Two domains were high risk as radiologists were not blinded to the reference. Applicability concerns’ assessment scored low risk in all domains. A total of 216 patients were scanned with exendin-4 tracers in 12 studies. 68Ga-DOTA-exendin-4 was the most used tracer, followed by 111In-DTPA-exendin-4. Other used chelators with 68Ga were NOTA and DOTAGA. SPECT images were also performed by labeling exendin-4 with 99mTc. Reported sensitivities ranged from 95% to 100% and specificities from 20% to 100%.

### Qualitative Assessment of the Selected Papers

Table 3 reports the main findings from the retrieved articles. The first report (by Christ et al.) of in vivo usage of exendin-4 dates back to 2009. ^111^In-DOTA-exendin-4 was injected into six patients with endogenous hyperinsulinemic hypoglycemia (EHH) symptoms. SPECT/CT was able to localize insulinoma lesions in all patients, proving a higher sensitivity than EUS when CT and MRI were considered the gold standard. Intraoperative radio-guided surgery could be done up to 14 days after injection and histopathology confirmed insulinomas in all cases [26]. In 2013, Christ et al. evaluated 25 patients who underwent both imaging and histological evaluation. Imaging was performed with ^111^In-DTPA-exendin-4 SPET, ceCT, and US. Early 4-h images showed a focal uptake in 20/25 patients. The other five patients underwent a late scan up to 72 h, which proved more conclusive. Sensitivity for insulinoma was 95%, with a positive predictive value (PPV) of 83%. Other lesions were categorized as nesidioblastosis and undetermined findings. CT/MRI showed a sensitivity of 47% and a PPV of 100%. An important limitation of the study was the low specificity for ^111^In-DTPA-exendin-4 (equal to 20%) [12]. Among the first studies performed, Antwi et al., in 2015, evaluated a small sample of five patients with both SPECT and PET exendin-4 tracers. Although only four patients underwent surgery and had histological confirmation of insulinomas, all five patients had a lesion uptake and were eligible for surgery [27]. In 2018, the same research team evaluated 52 patients with symptoms compatible with EHH who underwent PET and SPECT/CT with exendin-4 tracers as well as MRI. The main side effects of exendin-4 administration were nausea and vomiting within the first hour, especially for the SPECT tracer. Exendin-4 PET/CT showed the most impact on surgical planning and the surgery itself compared with the other two imaging techniques, making GLP-1R imaging useful for patient management [18]. In the same group of patients, insulinomas were part of the MEN-1 syndrome. Indeed, six patients with MEN-1 syndrome showed 37 lesions upon surgery. Exendin-4 PET/CT was able to identify 11/37 lesions, 10 of which were positive for insulinomas [19]. Pallavi et al. gathered eight patients with hyperinsulinemic hypoglycemia and neuroglycopenic symptoms. Three out of eight positive PET scans were compatible with insulinomas after histopathological analysis; overall, sensitivity was 75% [20]. Garg et al. retrospectively enrolled 14 patients who underwent abdominal ceCT, DOTATATE, and exendin-4 PET scans. The latter showed the highest sensitivity among the three imaging modalities (75% vs. 33.3% vs. 83.3%, respectively, for exendin-4 PET/CT, DOTATATE, and ceCT) [21]. Michalski et al. evaluated 10 patients with suspicion of insulinomas. Eight patients had a positive uptake on the exendin-4 PET scan, while the other two showed no focal pathological uptake. Therefore, in these latter two patients, the cause of hypoglycemia was considered of different origins, thus guiding medical treatment. Not all patients who had positive PET scans had surgery, therefore a statistical analysis was not performed [22]. Senica et al. developed a SPECT analog for GLP1R labeled with ^99m^Tc. Eight patients with suspected insulinoma were enrolled because of inconclusive conventional imaging. Multiple SPECT/CT images with exendin-4-based tracers were acquired at multiple time points showing sensitivity and specificity of 100% [23]. Kalff et al. enrolled 24 patients with EHH, and exendin-4 PET scan limitations were discussed. A false negative insulinoma on histopathology was due to the lack of GLP-1R expression in the lesion. This could then be studied with a SSR-2 receptor scan. False positive scans may be due to nesidioblastosis, as it is found to also express GLP-1R [14]. In 2022, Shah et al. evaluated three different imaging modalities such as ceCT, DOTATATE/DOTATOC, and exendin PET/CT in 36 patients. The main goal of the study was to assess how DOTA PET performed in EHH patients with morphological anomalies at ceCT. In this study, only 16/36 patients had an exendin PET retrospectively performed and, for this reason, it was difficult to make a solid statement regarding exendin-4 PET vs. DOTA-peptide PET vs. CECT. Nevertheless, the authors concluded that DOTA-PET was more sensitive for malignant lesions while exendin-4 performed better for benign lesions. This pattern was explained by the flip-flop phenomenon, meaning a higher expression of SSTR than GLP1-R in malignant lesions [24,30]. In 2016, a Chinese group with Luo et al. performed ^99m^Tc-HYNIC-TOC SPECT/CT and ^68^Ga-NOTA-exendin-4 PET/CT in 52 patients. Only 43 subjects underwent surgery, showing insulinomas in the histology. Exendin-4 PET/CT showed the highest sensitivity on both patient-based and lesion-based analyses (97.7% and 97.8%, respectively). ^99m^Tc-HYNIC-TOC showed the lowest sensitivity (19.5%). Overall sensitivity for other imaging methods was 84% for EUS, 74.4% for ceCT, and 56% for MRI [25]. Other Polish authors enrolled 11 patients with insulinomas who underwent exendin-4 labeled with ^99m^Tc and found a sensitivity and specificity of 100% for exendin-4 in benign lesions, while malignant lesions were positive only for somatostatin receptor scintigraphy [28]. The most recent study, dated February 2022, by Boss et al., compared exendin-4 with ^18^F-DOPA PET/CT in patients affected by congenital hyperinsulinism. Exendin-4 PET showed a higher sensitivity (100%) compared with DOPA PET (71%), with a high image quality difficult to identify [29].

## 4. Discussion

**Figure 2:**
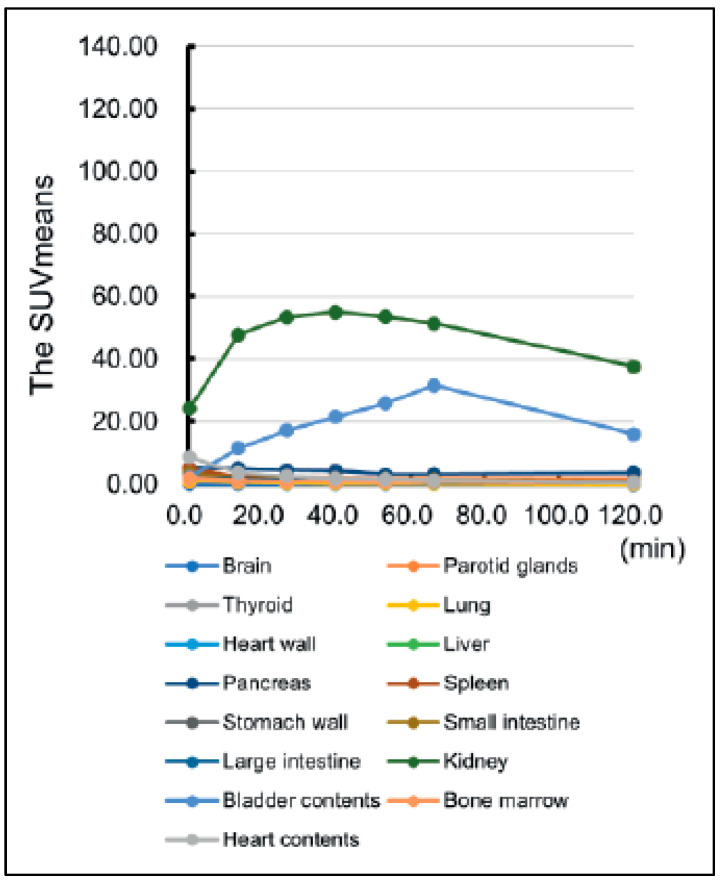
Time curve of mean standardized uptake value (SUVmean) in each organ from a cohort injected with [18F]FB(ePEG12)12-Exendin-4 [41].

**Figure 3:**
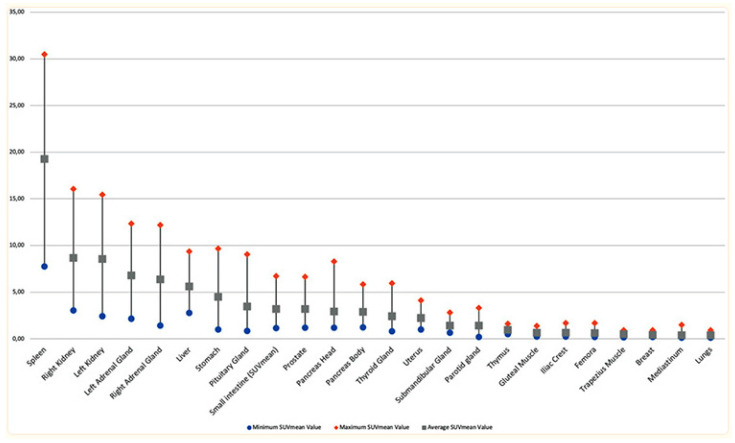
Organ uptake in normal subjects injected with 68Ga-DOTA-TATE [42].

As emerged from the present review, the currently available data are really promising regarding the use of exendin-4-based agents for PET/CT and SPECT imaging in the detection of insulinomas. Although limited, preliminary data are encouraging regarding the advantages of exendin-4 PET/CT compared with the standard imaging modality in defining the presence of insulinomas. However, it is difficult to talk about a better performance for a specific tracer because of the low number of studies available to date, the heterogeneity of the isotope and chelator used, as well as the technology implemented for imaging. However, the most data available belong to ^68^Ga-DOTA-exendin-4, owing to the easier supply from the ^68^Ge/^68^Ga generators and not requiring onsite cyclotron and with good sensitivity ranging from 75% to 94.6%. Studies that used ^99m^Tc provided a 100% sensitivity, although the low number of patients definitely played some role in this, inflating results. Nevertheless, the low radiation burden to patients and the better image quality in SPECT are definitely promising factors in insulinoma SPECT imaging. The opportunity to differentiate between malignant and benign lesions can be further supported by using different agents, such as labeling DOTA-SSTR. Some authors suggested that when exendin-4 imaging is not available, ^68^Ga-SSTR PET/CT could be the imaging method of choice, although it can still result in false negatives in nonfunctioning pancreatic neuroendocrine tumors due to a lack of SSTR receptors, mainly in cases of insulinoma [13]. The overall sensitivity, specificity, and detection rate for DOTATOC PET/CT were 58%, 89%, and 64%, respectively, for the identification of insulinomas [31]. Conversely, other studies showed greater sensitivity (85% and 90%) due to the inclusion of pathologically confirmed insulinoma lesions. A recent review by Shah et al. identified a pooled sensitivity and specificity of 94% and 83%, respectively, for ^68^Ga-exendin-4, significantly higher than other SPECT radiopharmaceuticals used to study and locate insulinomas [32]. In addition to the promising results of exendin-4-based agents for PET/CT and SPECT imaging in detecting insulinomas, it is important to consider the normal tracer uptake in the pancreas. In normal individuals, the pancreas typically demonstrates a low level of uptake for 68Ga-DOTA-exendin-4. Therefore, the identification of focal uptake in the pancreas is considered a strong indicator of insulinomas (Figure 2 and Figure 3). Nesidioblastotis, a rare cause of EHH recently associated by gastric bypass [33], is a frequent finding in exendin-4 imaging, independent of the chelator moiety in play, causing false positive reports, thus decreasing specificity of the method. Other false positive uptakes could be due to Brunner’s gland physiologic uptake, which is commonly seen in the proximal duodenum and can mimic insulinoma in the pancreas. On the other hand, when there is no evidence of focal uptake on the scintigraphic scan, with proof of biochemical recurrence, overlap uptake from surrounding structures must be kept in mind, as well as malignancy and lesions below camera resolution [34]. Recently, authors evaluated exendin-4 uptake in the brain of bariatric patients before surgery, as GLP1-R is also present in the brain and plays a role in appetite regulation. Although no significant uptake was reported, except for in the pituitary gland, this brings back the increased cases of nesidioblastosis in post-bariatric patients [35]. Studies showed that the N-terminal portion of exendin-4 (9–39) worked as an antagonist of GLP1-R and therefore it could be used as an imaging vector for insulinomas. Unfortunately, one radiopharmaceutical, ^125^I-BH-Ex(9–39)NH2, showed good pharmacokinetics in mice, with low kidney uptake and fast blood clearance [36]. Other antagonists such as [Lys40(DTPA-^111^In)]exendin(9–39), Lys27(Ahx-DOTA-^68^Ga)]Ex(9–39)NH2 and [Lys27(NODAGA-^68^Ga)]Ex(9–39)NH2 showed suboptimal binding capacity with the receptor as well as low tumor uptake [37,38]. Other authors compared the antagonist [Lys40(DTPA-^111^In)]exendin(9–39) with the agonists exendin-3 and exendin-4 and, despite the high affinity for the receptor, it showed low binding and minimal internalization, with low uptake in GLP1-R expressing tissue [38]. Vomiting is the most common side effect reported. When significant amounts of peptides are to be injected, this must stimulate the receptor and induce severe hypoglycemia. This can be prevented with a continuous infusion of glucose and this is where antagonist receptor imaging finds application [25]. Other more common tracers such as ^18^F-fluorodopa (^18^F-DOPA) have shown some application on insulinoma imaging, although very few data are available and, at first glimpse, they are less promising than GLP1-R imaging. Studies have shown a 50% sensitivity in detecting insulinoma lesions; this can go up to 73% when late scans are acquired and there is co-administration of carbidopa in order to reduce pancreas uptake to obtain a better target to background contrast [39,40]. Although the patient samples in these studies are small, they can still be compared with the QUADAS-2-assessed papers, with similar patient samples and better sensitivity reports. A recent study compared ^68^Ga-Somatostatin analogues (SSA) and ^18^F-DOPA diagnostic accuracy in neuroendocrine intestinal tumors. In a patient-based analysis, ^18^F-DOPA and ^68^Ga-SSA reported a pooled sensitivity of 83% and 88%, respectively, while in a per-lesion-based analysis, the pooled sensitivity was 95% and 82% in favor of ^18^F-DOPA.

### Future Prospectives

**Figure 4:**
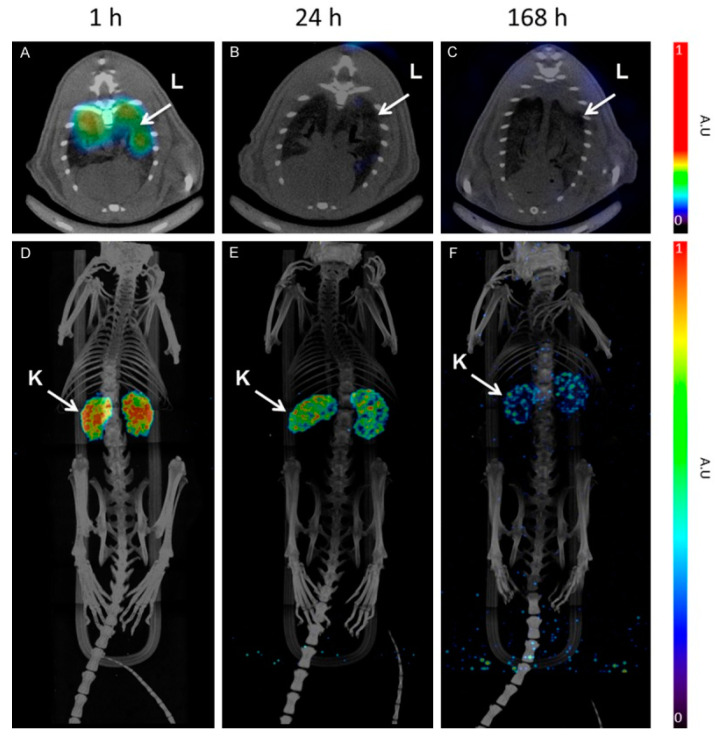
SPECT/CT images of [177Lu]-DO3A-VS-Cys40-Exendin-4 at 1, 4, and 168 h. Lungs are visible 1 h p.i. and show fast clearance (**A**–**C**). MIP images (**D**–**F**) of whole body showing mainly kidney uptake [47].

One of the most relevant features of radionuclide-based imaging is its capability to identify tumor-associated biomarkers that can be exploited for molecularly targeted therapies, thus combining diagnosis and therapy in a unique approach, namely “theranostics or theranostics”. In this perspective, exendin-4 has been employed in some theranostic applications [43,44]. Although, as we have seen, ^111^In-DTPA-exendin-4 has imaging properties because of its γ-ray emissions, low-energy Auger electrons might provide cytotoxic damage to the nearby DNA, as was shown by some authors in mouse models. The main limitation of ^111^Indium is posed by its high radiation dose, especially to the kidney, leading to renal radiation damage and chronic renal failure [45]. Kidney uptake, as it is not due to GLP1-R expression, is caused by glomerular filtration followed by tubular resorption, leading to increased bioretention in the renal parenchyma [38]. To the best of our knowledge, there are no papers comparing theranostic studies with DOTA peptides and exendin tracers, probably owing to the different uptake mechanism and the different tumor targets of the tracers, as the first targets malignant NEN and the other insulinomas. Another proposed tracer is [Lys40(Ahx-DTPA-^111^In)NH2], with tumor shrinkage in mice up to 94%, but still owing to the kidney radiation from ^111^Indium [45]. Dutch authors proposed the injection of gelofusine to reduce the kidney dose, since it reduces the renal accumulation of radiolabeled peptides, without affecting the pancreas lesion uptake [46]. Dosimetric studies conducted using ^177^Lu-DO3A-VS-Cys40-exendin-4 in mice and in a human estimation on a patient using ^68^Ga-DO3A-VS-Cys40-exendin-4 confirmed the need to protect the kidneys from radiation damage (Figure 4) [47,48]. Although still not investigated, the coupling of iodine ^123^I in SPECT and ^124^I in PET with the radiotherapeutic ^131^I could bypass kidney toxicity, but we have yet to study whether gastric and colonic adverse effects are present. ^188^Rhenium could also see its application in theranostics, as the more widespread use of ^99m^Tc in nuclear medicine facilities could facilitate the theranostic coupling. Intraoperative γ probes have seen application in exendin-4 imaging, but the development of fluorescence-guided surgery for insulinomas has also seen progress, with already developed various exendin-4 fluorescent tracers [49,50]. These probes can detect fluorophores in the far-red wavelength (800 nm) and have the advantage, compared with γ probes, of theoretically causing less damage to the structures in play. Photodynamic therapy could also be an alternative in tumor ablation. It consists of photosensitizer administration followed by lighting of the target lesion with near-infrared wavelengths. In short, the photosensitizer in the ground state is activated and, by emission of energy, is converted back to the ground state. This process is responsible for tumor cell killing [51,52]. The main advantage of this technique is to reduce excretory organ toxicity due to greater tracer uptake, which plays a very much limiting role with ^111^Indium in the kidneys. Nevertheless, the indication for a fluorescent-guided intraoperative procedure and/or therapy should be preceded by functional imaging, as malignant and benign insulinomas have different expressions of receptor, higher SSTR, and lower GLP1-R in malignant lesions and vice versa in benign lesions.

## 5. Conclusions

Clinical data, although very promising, are still not enough to include exendin-4 functional imaging into the diagnostic guidelines for insulinomas, as endoscopic ultrasound still performs most of the job when correlated with patient history. When lesion location is hard to reach, morpho-structural and functional imaging with DOTA-peptides and extending-4 find a role in the diagnosis of benign and malignant insulinomas, helping the clinician and the surgeon to better localize and enucleate the lesion. Even though insulinomas are the most common among rare tumors, exendin-4 will bring innovation to neuroendocrine neoplasm imaging, as theranostics make strides within the clinical setting when patients are averse to surgery or are inoperable.

## Figures and Tables

**Table 1:** QUADAS-2 risk of bias assessment.

Authors, Year of Pub (Ref)	Risk of Bias Assessment			
	Patient Selection	Index Test	Reference Standard	Flow and Timing
Antwi et al., 2018 [18]	Low	High	Low	Low
Antwi et al., 2019 [19]	Low	Low	Unclear	Low
Pallavi et al., 2019 [20]	Low	Unclear	Unclear	Low
Garg et al., 2020 [21]	Low	Low	Unclear	Low
Michalski et al., 2020 [22]	Low	Unclear	Unclear	Low
Kalff et al., 2021 [14]	Low	Low	Unclear	Low
Senica et al., 2020 [23]	Low	Unclear	Unclear	Low
Shah et al., 2022 [24]	Low	Low	Unclear	Low
Crist et al., 2013 [12]	Low	Low	Low	Low
Luo et al., 2016 [25]	Low	Unclear	Unclear	Low
Crist et al., 2009 [26]	Low	Unclear	Unclear	Low
Antwi et al., 2015 [27]	Low	Unclear	Low	Low
Sowa-Staszczak et al., 2013 [28]	Low	Unclear	Unclear	Low
Boss et al., 2022 [29]	Low	High	Unclear	Low

**Table 2:** QUADAS-2 applicability concerns’ assessment.

Authors, Year of Publication (Ref)	Applicability Concerns’ Assessment		
	Patient Selection	Index Test	Reference Standard
Antwi et al., 2018 [18]	Low	Low	Low
Antwi et al., 2019 [19]	Low	Low	Low
Pallavi et al., 2019 [20]	Low	Low	Low
Garg et al., 2020 [21]	Low	Low	Low
Michalski et al., 2020 [22]	Low	Low	Low
Kalff et al., 2021 [14]	Low	Low	Low
Senica et al., 2020 [23]	Low	Low	Low
Shah et al., 2022 [24]	Low	Low	Low
Crist et al., 2013 [12]	Low	Low	Low
Luo et al., 2016 [25]	Low	Low	Low
Crist et al., 2009 [26]	Low	Unclear	Unclear
Antwi et al., 2015 [27]	Low	Unclear	Low
Sowa-Staszczak et al., 2013 [28]	Low	Unclear	Unclear
Boss et al., 2022 [29]	Low	Low	Low

**Table 3:** Eligible studies’ characteristics.

Author, Ref	Year of Publication	Country	Tracer	N of pts	Accuracy	Sensitivity	Specificity
Christ et al., [26]	2009	Switzerland	^111^In-DOTA-exendin-4	6	Not explicit	Not explicit	Not explicit
Christ et al., [12]	2013	Switzerland	^111^In-DTPA-exendin-4	25	Not explicit	95%	20%
Sowa-Staszczak et al., [28]	2013	Poland	[Lys40(Ahx-HYNIC-^99m^Tc/EDDA)NH2]-exendin-4	11	Not explicit	100%	100%
Antwi et al., [27]	2015	Switzerland	^111^In-DOTA-exendin-4, ^68^Ga-DOTA-exendin-4	5	Not explicit	Not explicit	Not explicit
Luo et al., [25]	2016	China	^68^Ga-NOTA-exendin-4, ^99m^Tc-HYNIC-TOC	52	Not explicit	97.7%	Not explicit
Antwi et al., [18]	2018	Switzerland	^68^Ga-DOTA-edendin-4; ^111^In-DOTA-exendin-4	52	93.9% PET/CT; 67.5% SPECT/CT	94.6% PET/CT; 68.5% SPECT/CT	Not explicit
Senica et al., [23]	2020	India	[99mTc]EDDA)NH2]-exendin-4	8	Not explicit	100%	100%
Antwi et al., [19]	2019	Switzerland	^68^Ga-DOTA-edendin-4	52	94.6% PET/CT	84.6% PET/CT	100% PET/CT
Pallavi et al., [20]	2019	India	^68^Ga-DOTA-edendin-4	8	Not explicit	75% PET/CT	Not explicit
Garg et al., [21]	2020	India	^68^Ga-NODAGA-edendin-4; ^68^Ga-DOTATATE	14	Not explicit	83.3% exendin-4; 33.3% DOTATE	Not explicit
Michalski et al., [22]	2020	India	^68^Ga-DOTA-edendin-4	10	Not explicit	Not explicit	Not explicit
Kalff et al., [14]	2021	Australia	^68^Ga-DOTA-exendin-4	25	84%	Not explicit	Not explicit
Boss et al., [29]	2022	Netherlands	^68^Ga-NODAGA-exendin-4	19	Not explicit	100%	Not explicit

## Data Availability

Not applicable.
